# Component resolved diagnosis: when should it be used?

**DOI:** 10.1186/2045-7022-4-28

**Published:** 2014-09-08

**Authors:** Olga Luengo, Victòria Cardona

**Affiliations:** 1 Allergy Section, Department of Internal Medicine, Hospital Vall d’Hebron, Barcelona, Spain; 2 Allergy Research Group, Institut de Recerca Vall d’Hebron, Barcelona, Spain

**Keywords:** Molecular allergy, Component resolved diagnosis, Allergen molecules, Allergen microarray, Polysensitization, Immunotherapy

## Abstract

The knowledge on molecular allergy diagnosis is continuously evolving. It is now time for the clinician to integrate this knowledge and use it when needed to improve the accuracy of diagnosis and thus provide more precise therapeutic and avoidance measures. This review does not intend to comprehensively analyze all the available allergen molecules, but to provide some practical clues on use and interpretation of molecular allergy diagnosis. The potential role of component resolved diagnosis in circumstances such as the indication of allergen immunotherapy, pollen polysensitization, food allergy, latex allergy or anaphylaxis, is assessed. Interpreting the information provided by molecular allergy diagnosis needs a structured approach. It is necessary to evaluate single positivities and negativities, but also to appraise “the big picture” with perspective.

## Introduction

Nearly 15 years after the concept of component-resolved diagnosis was first proposed [1], the amount of knowledge on the molecular allergy diagnosis is continuously evolving. Several comprehensive and extensive reviews on component resolved diagnosis (CRD) have been published, and their use and limitations in clinical practice proposed [2-4]. It is now time for the clinician to integrate this knowledge and use it when needed to improve the accuracy of diagnosis and thus provide more precise therapeutic and avoidance measures.

However, even after a thorough study of the literature the interpretation of molecular allergy diagnosis can initially seem very complex. So, there is an unmet need for educational programs on the use and interpretation of molecular diagnosis. Among other initiatives, our group has been developing a practical training program on molecular allergy diagnosis for the last four years with attendees from Spain and Portugal. With the WAO-ARIA-GALEN consensus on molecular diagnosis [4] as the framework, clinicians attending these workshops developed a proposal on “when to use CRD” in their routine care that can be summarized as shown in Table 1, which has been used as a guide for this paper. This review does not intend to comprehensively analyze all the available allergen molecules, or all the specific aspects of food, respiratory, venom or latex allergy, but to provide some practical clues on use and interpretation of molecular allergy diagnosis.

**Table 1:** Proposals on when to use CRD

**Circumstances of potential increase of allergy diagnosis accuracy by CRD**	
• Indication of allergen immunotherapy	o Inhalant oligo/monosensitization
	o Pollen polysensitization
	o Hymenoptera venom allergy
• Anaphylaxis	o Cofactor-enhanced food-dependent anaphylaxis
	o Delayed red meat anaphylaxis
	o Idiopathic anaphylaxis
• Latex allergy	
• Polysensitization	o Pollen and plant food
• Food allergy	o Risk assessment
	o Identification of unanticipated allergen triggers

### Indication of specific immunotherapy

**Figure 1:**
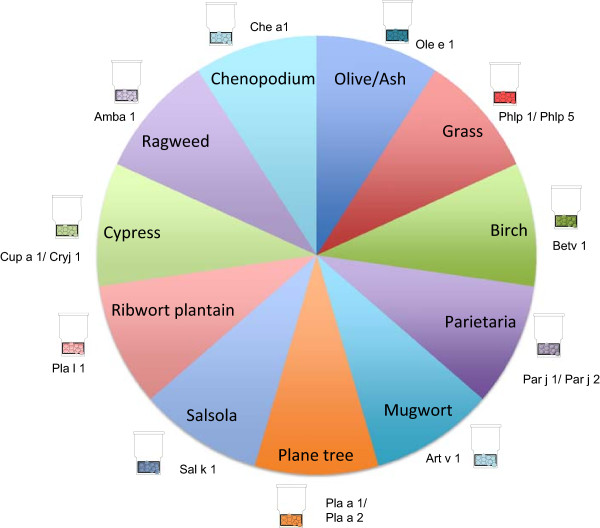
Pollen species-specific allergens.

**Figure 2:**
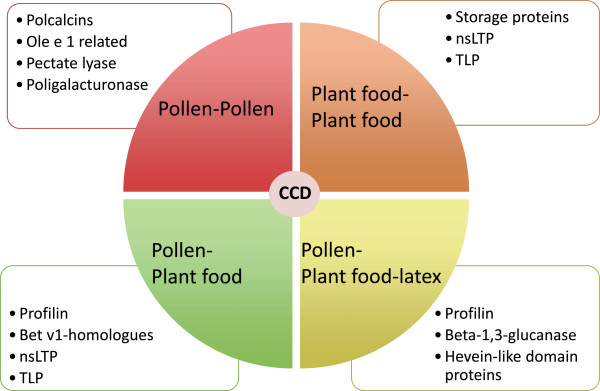
**Cross-reactive allergens.** CCD: Cross-reactive carbohydrate determinants; nsLTP: Non-specific lipid transfer proteins; TLP: thaumatin-like proteins.

The first premise for the prescription of immunotherapy based on CRD is the assessment of IgE positivity to genuine versus cross-reactive allergens (Figures 1 and 2).

### Inhalant oligo/monosensitization

Precise identification of relevant sensitizers in the case of pollen-allergic patient’s mono or oligosensitized to pollens with no overlapping pollen season can be achieved by conventional diagnosis with complete pollen extracts. In most cases patients are sensitized to major pollen allergens (e.g. Ole e 1, Bet v 1, Phl p 1/Phl p 5), but this may not be the case in areas with high pollen loads, for example to olive pollen in the south of Spain, where Ole e 7 and Ole e 9, currently considered as minor allergens, can be the major sensitizers [5]. When prescribing specific immunotherapy in areas with high frequency of sensitization to “minor allergens”, molecular diagnosis may be of special interest, since commercial extracts for immunotherapy are well standardized only for major allergens. Thus, patients with sensitization to minor allergens alone may likely not receive sufficient amounts of allergen to achieve a successful outcome from allergen immunotherapy (AIT), or even worse, will experience adverse reactions when the concentrations of this minor allergens present in the extract are high [6].

Another scenario in which molecular allergy would help to decide the correct indication of AIT would be dog dander allergy. Unlike cat allergy, almost all attributable to sensitization to its major allergen Fel d 1, the sensitization profile in case of dog allergy is more heterogeneous [7]. In Spanish populations, Can f 5 is a major allergen, with reported sensitizations of 70% of dog allergic patients [8]. In our series, Can f 1, 2 and 3 are minor allergens, while Can f 5 is responsible for up to 67% of sensitizations and, importantly 37% of our patients are not sensitized either to Can f 1, 2 or 3 but only to Can f 5 [9]. There is a high variability between commercial dog extracts regarding their allergen contents [10], and Can f 5 is poorly represented. It would not seem appropriate to indicate specific immunotherapy to dog extract in patients monosensitized to Can f 5 until this (and possibly other) major allergen content is guaranteed in the therapeutic extract.

### Pollen polysensitization

Unfortunately mono/oligosensitized patients are more and more scarce, at least in adults whose diagnostic complexity increases with polysensitization [11]. Although most studies on the relevance of CRD in complex pollen areas have been performed in the south of Europe, polysensitization to respiratory allergens is also seen in the north. This has been shown by data of the European Community Respiratory Health Survey (ECRHS) where 12.8% to 25.3% of patients were polysensitized [12]. This fact has important implications when considering the prescription of immunotherapy. In recent large clinical trials, single-allergen immunotherapy with grass pollen extract has proved to be as safe and effective for that specific allergy both in polysensitized as in monosensitized patients [13,14], provided that the allergen extract administered matches the patient’s most relevant sensitization.

CRD provides the information on patient specific allergen sensitization to drive the selection of the immunotherapy extract [15], conceptually “component-resolved treatment”. AIT would be appropriately prescribed if sensitization to the species-specific allergens is confirmed, while in case of selective recognition of cross-reactive allergens, like profilins or CCD, the indication of AIT is arguable. Cross-reactive allergens seem to have limited clinical relevance and their content in AIT extracts is usually not quantified. Also, in the case of sensitization to the crude extract (SPT and/or positive sIgE), AIT indication would be arguable if all components are negative, since the extracts would be unlikely to contain the sensitizing molecule.

Proving the importance of a CRD-driven immunotherapy prescription, three prospective studies, including adult and pediatric population, have recently shown that the incorporation of CRD results alters initial AIT prescription in approximately half of the patients [16-18]. However, there is still a gap between the current possibility of using a predefined AIT preparation and the complexity of sensitization at the population level, since the patients sIgE profiles are highly heterogeneous depending on the geographical area and the allergen source [19,20]. Also, there is a need to adequately evaluate in prospective studies if CRD-guided patient selection results in improved efficacy of immunotherapy.

Since the first proposal of Valenta [1], few articles have been published on how to use CRD results for the optimal selection of immunotherapy. Very recently Douladiris et al. have proposed a comprehensive and practical algorithm regarding component-resolved diagnostic work-up for pollen AIT candidates in southern Europe [21].

### Hymenoptera venom immunotherapy

Molecular diagnosis can also improve the selection of patients for hymenoptera venom immunotherapy (VIT). The diagnosis of hymenoptera venom allergy should be performed using non-glycosilated allergens to avoid false-positive sIgE results due to cross-reactive carbohydrates. Commercially available species-specific major allergens without CCD include Api m 1 from bee venom (phospholipase), Ves v 1 (phospholipase) and Ves v 5 (antigen 5) from Vespula venom and Pol d 5 (antigen 5) from Polistes dominulus venom.

Antigen 5 and phospholipase of both *Vespula vulgaris* and *Polistes dominulus* help discriminate between true genuine allergy and serological cross-reactivity in cases of double positivity to traditional sIgE and venom skin tests to both vespids [22]. Ebo et al. [23] also propose the use of CRD with rVes v 1 and rVes v 5 in patients with double-positive sIgE to yellow jacket and honey bee venom, discrepant sIgE and venom skin test results, as well as patients with negative traditional sIgE and skin tests. Müller et al. [24] recommend the indication of VIT to both bee and *Vespula* venoms in patients with double positivity of sIgE to whole venoms, and sIgE to Api m 1, Ves v 1 and Ves v 5. If only positive to Api m 1, VIT should be indicated only with bee venom. In patients with sIgE only to Ves v 1 and Ves v 5, VIT with *Vespula* venom would be certainly indicated. rVes v 5 has also proved to facilitate diagnosis of hymenoptera venom allergy in patients with negative sIgE to wasp venom [25]. In the case of honeybee venom allergy it has been recently published that a broader panel of CCD-free honey bee venom allergens, including rApi m 2, rApi m 3, nApi m 4, rApi m 5, and rApi m 10, improves diagnostic sensitivity compared with use of rApi m 1 alone [26].

## Anaphylaxis

### Cofactor-enhanced food-dependent anaphylaxis

Epidemiological data show that cofactors (exercise, NSAIDs, alcohol, etc.) are relevant in up to 39% of all food-dependent anaphylactic reactions in adults [27]. Wheat dependent exercise-induced anaphylaxis (WDEIA) is the best characterized of these syndromes, classically related to omega-5-gliadin sensitization. Recently it has been reported that, at least in the Mediterranean population nsLTP accounts for the majority of cofactor-enhanced food allergy (CEFA) [28,29] mainly related with vegetables, nuts and cereals. Even in cases of WDEIA reactions, positivity to nsLTP in the absence of omega-5-gliadin sensitization has been reported [30]. Therefore, at least in southern Europe, patients with a history consistent with CEFA anaphylactic reactions should be tested for sIgE to nsLTP (mainly Pru p 3, but also to Tri a 14) and to omega-5-gliadin. Other underlying sensitizations may be relevant in some populations.

### Red meat delayed anaphylaxis

When evaluating a patient with a history of delayed onset anaphylaxis 3–6 h after ingestion of mammalian food products (e.g., beef and pork), sIgE against galactose-α-1,3-galactose (α-gal) should be performed [31]. Before the identification of the allergen responsible for this syndrome, because of the delay of symptoms after ingestion of meat products, the frequent negative SPT responses and the good tolerance to other meats like turkey, these types of anaphylaxis have been wrongly classified as idiopathic [32]. It has been suggested that tick bites are the cause of IgE antibody responses to α-gal and it is recommended to reassess sIgE levels every 8 to 12 months as they tend to decrease over time, and some patients have been able to tolerate mammalian meat again after avoiding additional tick bites for 1 to 2 years [33].

### Idiopathic anaphylaxis

Although idiopathic anaphylaxis involves a small proportion of patients with anaphylaxis, the clinical implications are highly significant. The inability to identify a cause prevents from usual anaphylaxis interventions such as avoidance measures, specific education and modification of risk.

To date, only one study has addressed the question whether the ISAC allergy array would add diagnostic value in patients with idiopathic anaphylaxis. Heaps et al. [34] performed an ISAC-103 test (Thermo Fischer Scientific, Uppsala, Sweden) to 110 patients with a diagnosis of idiopathic anaphylaxis in UK and found new allergenic sensitizations in half of the patients studied, which in 20% of the cases were identified as the cause of the anaphylaxis with a high likelihood (although it was only reassessed in 50% of those patients). Omega-5-gliadin and shrimp allergens accounted for 45% of the previously unrecognized sensitizations. Other newly identified allergens related to the anaphylaxis were seed storage proteins, nsLTP and latex allergens. We must bear in mind that some molecules are poorly represented in allergen extracts, and therefore the sensitivity of conventional diagnostic tests (SPT, sIgE) will not allow a diagnosis.

Therefore, the performance of a multiplex CRD and sIgE to α-gal, when available, would be very helpful in the assessment of idiopathic anaphylaxis. If positive, it may orientate on the triggering allergen; if negative, a non-IgE mediated mechanism underlying the anaphylaxis may be more likely.

## Latex allergy

Correct identification of latex-sensitized patients with true latex allergy is of major importance as these patients have an increased risk for potentially severe reactions during medical procedures. On the other hand, identification of irrelevant latex-sensitization due to cross-reactive allergens would avoid a wrong diagnosis of latex allergy, prevent unnecessary latex-avoidance measures and reduce healthcare costs.

Latex allergens Hev b 1, Hev b 3, Hev b 5 and Hev b 6 are currently considered markers of genuine latex sensitization. On the other hand, studies on CRD of latex allergy have consistently shown that the majority of latex-sensitized persons, asymptomatic upon latex exposure, have a profilin sensitization with monosensitization to Hev b 8 [35,36]. Typically, these patients have a positive sIgE against latex, but are negative in SPT and do not show latex-specific symptoms upon contact with latex-containing material [37]. Haeberle et al. [38] and Quercia et al. [39] have reported that these patients can undergo major surgery in normal surgical setting without any consequences. Thus, Hev b 8 has been proposed as a marker of asymptomatic latex sensitization.

In the particular case of hymenoptera venom allergy with positive sIgE to latex, sensitization to CCD should be ruled out since insect venoms and latex share IgE-binding CCD responsible for non-clinically relevant positive serological test to commercial latex extract [40].

As a practical approach, in case of unequivocal clinical history of latex allergy with positive NRL-SPT and/or sIgE it may not be necessary to perform a CRD study since no association between allergens and severity of reactions has been identified so far.

Although cross-reactivity between several latex allergens (Hev b 5, Hev b 6, Hev b 7, Hev b 11, Hev b 13,…) and plant-food allergens has been described to explain the so called “latex-fruit syndrome”, to date there are no risk-assessment studies evaluating molecular sensitization profiles and clinical food-allergy in latex allergic patients.

### Polysensitization to inhalant and food allergens

One of the biggest challenges for the allergist is to confront the patient with positive SPT to several pollen and food allergens. In this scenario CRD may be of major usefulness, improving the resolution of conventional diagnosis by adding information on the genuine primary sensitizers to distinguish them from sensitization due to cross-reactivity [41] (Figures 1 and 2). With regard to poly-pollen sensitization, this information may be relevant for prescribing AIT as discussed before.

Polysensitization to animal dander (cat, dog and horse) can in part be explained by cross-reactive lipocalins and albumins [7]. Can f 6 (dog lipocalin) is a likely candidate for cross-species sensitization with cat (Fel d 4) and horse (Equ c 1) [42] with clinical relevance [43]. Serum albumin is also implicated in cross-reactivity in the so-called cat-pork syndrome, where patients developing a cat serum albumin IgE response react upon pork meat ingestion [44].

Allergic reactions to fruits and vegetables can result from a primary sensitization to food or to inhalant allergens. Usually, cross reactivity is attributable to labile allergens (e.g., PR-10 and profilins) and associated with mild oral reactions [45], while heat and proteolysis-resistant allergens that primary sensitize through the oral route, are associated with systemic reactions in addition to local reactions (e.g. seed storage proteins and nsLTP) [2]. Sensitization to CCD in food or venoms does not have remarkable clinical relevance, and the primary sensitization may derive from either pollen or venoms. [46] Since purified native allergens may express carbohydrates (while recombinants do not), determination of sIgE to MUXF3 (a type of CCD) should be performed to rule out irrelevant sensitization to CCD in case of positive sIgE to purified native glycosilated allergens without clinical symptoms [47].

## Food allergy

### Risk assessment

Since the first study on CRD in apple allergy across Europe [48], where it was demonstrated that sensitization to apple nsLTP (Mal d 3) was associated with a 7 fold risk of anaphylaxis compared to sensitization apple Bet v 1 homologue (Mal d 1), nsLTP have been considered markers of severe allergic reactions. However, studies on patterns of nsLTP sensitization in Mediterranean patients have shown that the clinical expression is variable, ranging from asymptomatic sensitization to severe anaphylaxis [49,50], possibly modulated by pollen allergen co-sensitization and the presence of cofactors [51]. A predictive pattern of clinical expression in nsLTP-sensitized patients has not yet been elucidated.

Bet v 1 homologues are considered markers of mild allergic reactions to fruits and vegetables due to cross-reactivity with birch pollen. However, although not frequent, some anaphylactic reactions to apple in patients sensitized to PR10-proteins have been reported [52]. In the particular case of soya allergy, Gly m 4 (the Bet v 1 related allergen in soya) has been related to severe, generalized symptoms [53].

Seed storage proteins from nuts and soya have been associated with higher risk of severe allergic reactions [54]. In the case of peanut, Ara h 2 seems to be the best predictor of peanut allergy, reducing the need for peanut challenges by at least 50% [55]. Altogether, Ara h 1, Ara h 2 and Ara h 3 have been associated with severe symptoms, although anaphylactic reactions have been described in patients negative for these allergens [56].

Sensitization to Cor a 9 and Cor a 14 have been reported to be highly specific for hazelnut allergic patients with objective symptoms in DBPCFCs and proposed as markers for a more severe hazelnut allergic phenotype [57]. Similarly, in patients with soybean allergy, Gly m 5 and Gly m 6 have been proposed as potential markers for severe allergic reactions [58].

**Figure 3:**
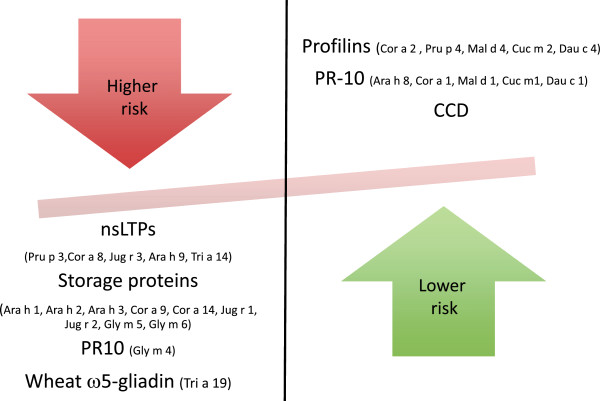
**Allergens associated to higher versus lower risk of anaphylaxis.** CCD: Cross-reactive carbohydrate determinants; nsLTP: Non-specific lipid transfer proteins.

Altogether, CRD may be a useful tool for stratifying patient’s risk for severe reactions but it is important to bear in mind that the risk of developing anaphylaxis depends not only on the allergen sensitization pattern, but also on the avidity and affinity of immunoglobulins to bind the allergen, the route of application, characteristics of the allergen and the presence of cofactors [27]. Figure 3 depicts those allergens that have been associated to higher versus lower risk of anaphylaxis.

### Identification of unanticipated allergen triggers

The sensitization profile also has major implications regarding the scope of types of plant foods that may trigger symptoms. Sensitization to seed storage proteins will limit allergic symptoms to nuts and occasionally to legumes [59], while sensitization to PR-10, nsLTP [60] or thaumatin-like proteins (TLP) [61] is frequently associated with symptoms with multiple fruits, vegetables, etc. However, currently there are no markers to predict the natural history; these patients should be followed-up and informed about the potential future reactions, although there is no evidence to advice avoidance.

### When to use singleplex or multiplex CRD

**Figure 4:**
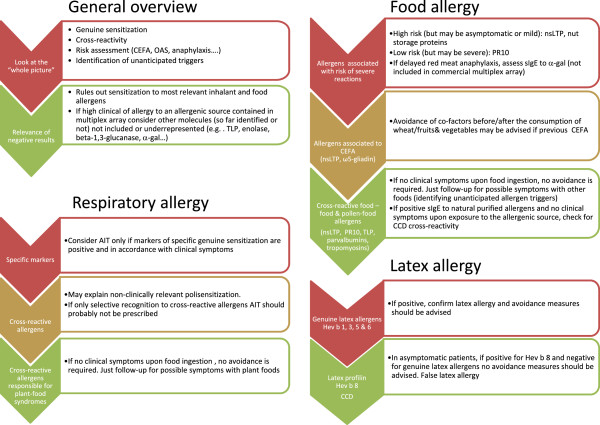
**Multiplex CRD interpretation flow-chart.** AIT: Allergen immunotherapy; CCD: Cross-reactive carbohydrate determinants; CEFA: cofactor enhanced food allergy; NSAID: non-steroidal anti-inflammatory drugs; nsLTP: Non-specific lipid transfer proteins; OAS: oral allergy syndrome; PR-10: pathogenesis-related protein family 10 (Bet v 1- homologues); TLP: thaumatin-like proteins.

Single allergens for CRD can be either recombinant (r) or natural purified (n) and sIgE can be measured either in singleplex or multiplex platforms. Depending on the aim of the CRD (guidance of AIT, polysensitization, latex allergy, etc.), the availability in each country and the complexity of the case, the clinician will choose one or the other. In general, for complex cases of multiple sensitizations to respiratory and food allergens as well as for idiopathic anaphylaxis study, a multiplex CRD should be performed. The clinician should be aware of benefits and pitfalls of molecular multiplex platforms before initiating a study. Multiplex assays allow up to 112 allergen assays in parallel with the use of very low serum quantities and no interference from very high total IgE but are less sensitive than singleplex assays and less appropriate for monitoring sensitization [4]. A guideline to help the allergy specialist to interpret multiplex molecular allergy diagnosis is proposed (Figure 4).

## Conclusions

**Figure 5:**
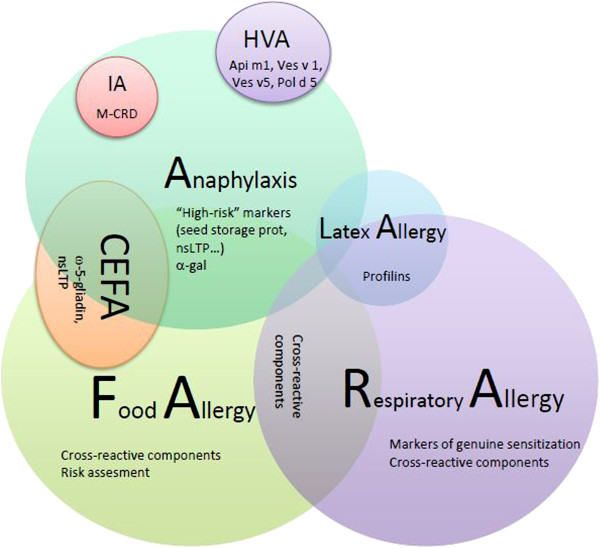
**Hypothetical scheme representing the potential use of CRD.** Represented by spheres are the allergic conditions in which molecular diagnosis may be of potential value, illustrating the potential overlap between different clinical reactions. Recommended components to be tested are listed; multiplexed CRD would be of special interest in idiopathic anaphylaxis and polysensitized patients. CEFA: cofactor-enhanced food allergy; HVA: hymenoptera venom allergy; IA: idiopathic anaphylaxis; M-CRD: multiplex CRD; nsLTP: non-specific lipid transfer proteins.

The vast information provided by molecular allergy needs a structured approach in order to be adequately interpreted. There is a need to evaluate single positivities and negativities, but also to appraise “the big picture” with perspective (Figure 5). When making decisions on this information, one has to bear in mind what is included in the tests and what is missing. It is needed that we understand that not all allergenic sources are present in the available arrays, but that the most important allergenic protein families are. This is relevant both when searching for a culprit allergen as when ruling out possible causes of certain reactions.

## Abbreviations

AIT: Allergen immunotherapy; CCD: Cross-reactive carbohydrate determinants; CEFA: Cofactor-enhanced food allergy; CRD: Component resolved diagnosis; MA: Molecular allergy; nsLTP: Non-specific lipid transfer proteins; OAS: Oral allergy syndrome; TLP: Thaumatin-like proteins.

## Competing interests

The Allergy Research Group, to which OL and VC belong, receives research funds from Thermo Fischer Scientific, Spain.

## Author’s contributions

All authors have equally collaborated in the design and drafting of this review. All authors read and approved the final manuscript.
